# Development of Cerium Oxide-Laden GelMA/PCL Scaffolds for Periodontal Tissue Engineering

**DOI:** 10.3390/ma17163904

**Published:** 2024-08-07

**Authors:** Sahar Aminmansour, Lais M. Cardoso, Caroline Anselmi, Ana Beatriz Gomes de Carvalho, Maedeh Rahimnejad, Marco C. Bottino

**Affiliations:** 1 Department of Cariology, Restorative Sciences and Endodontics, School of Dentistry, University of Michigan, 1011 N. University Avenue, Ann Arbor, MI 48109, USA; amsahar@umich.edu (S.A.); lais.cardoso@unesp.br (L.M.C.); caroline.anselmi-oliveira@unesp.br (C.A.); anab.gomes@hotmail.com (A.B.G.d.C.); rmaedeh@umich.edu (M.R.); 2 Department of Dental Materials and Prosthodontics, School of Dentistry, São Paulo State University (UNESP), 1680 Humaitá Street, Araraquara 14801-903, SP, Brazil; 3 Department of Morphology and Pediatric Dentistry, School of Dentistry, São Paulo State University (UNESP), 1680 Humaitá Street, Araraquara 14801-903, SP, Brazil; 4 Department of Dental Materials and Prosthodontics, School of Dentistry, São Paulo State University (UNESP), 777 Eng. Francisco Jose Longo Avenue, São José dos Campos 12245-000, SP, Brazil; 5 Department of Biomedical Engineering, College of Engineering, University of Michigan, Ann Arbor, MI 48109, USA

**Keywords:** periodontium, gelatin methacryloyl, polycaprolactone, cerium oxide, electrospinning

## Abstract

This study investigated gelatin methacryloyl (GelMA) and polycaprolactone (PCL) blend scaffolds incorporating cerium oxide (CeO) nanoparticles at concentrations of 0%, 5%, and 10% *w/w* via electrospinning for periodontal tissue engineering. The impact of photocrosslinking on these scaffolds was evaluated by comparing crosslinked (C) and non-crosslinked (NC) versions. Methods included Fourier transform infrared spectroscopy (FTIR) for chemical analysis, scanning electron microscopy (SEM) for fiber morphology/diameters, and assessments of swelling capacity, degradation profile, and biomechanical properties. Biological evaluations with alveolar bone-derived mesenchymal stem cells (aBMSCs) and human gingival fibroblasts (HGFs) encompassed tests for cell viability, mineralized nodule deposition (MND), and collagen production (CP). Statistical analysis was performed using Kruskal–Wallis or ANOVA/post-hoc tests (α = 5%). Results indicate that C scaffolds had larger fiber diameters (~250 nm) compared with NC scaffolds (~150 nm). NC scaffolds exhibited higher swelling capacities than C scaffolds, while both types demonstrated significant mass loss (~50%) after 60 days (*p* < 0.05). C scaffolds containing CeO showed increased Young’s modulus and tensile strength than NC scaffolds. Cells cultured on C scaffolds with 10% CeO exhibited significantly higher metabolic activity (>400%, *p* < 0.05) after 7 days among all groups. Furthermore, CeO-containing scaffolds promoted enhanced MND by aBMSCs (>120%, *p* < 0.05) and increased CP in 5% CeO scaffolds for both variants (>180%, *p* < 0.05). These findings underscore the promising biomechanical properties, biodegradability, cytocompatibility, and enhanced tissue regenerative potential of CeO-loaded GelMA/PCL scaffolds for periodontal applications.

## 1. Introduction

Periodontal disease is a major public health concern characterized as a multifactorial condition with contributions from genetic predispositions, microbial activity, and environmental influences [1]. This inflammatory condition initially targets the soft tissue, specifically the gingiva surrounding the teeth, and may advance to affect the bone and periodontal ligament. These structures anchor the teeth to the alveolar bone, potentially resulting in tooth mobility and eventual loss [2].

Periodontal regeneration, a multifaceted process involving infection and inflammation control, stem/progenitor cell recruitment, cell proliferation and differentiation promotion, and new tissue formation, poses a significant challenge in periodontal research and clinical practice. Traditional approaches for periodontal disease management focus on eliminating infection and reducing inflammation but fall short of achieving full tissue regeneration [3]. Current treatments for advanced stages of periodontal disease are limited because there are no commercially available materials capable of supporting tissue healing and coordinating the regeneration of periodontal tissues. Therefore, the imperative development of alternative approaches, such as membranes for guided tissue regeneration (GTR), is paramount [3].

Periodontal tissue engineering approaches endeavor to enhance patient care by facilitating the restoration of damaged and lost tissues through various strategies, including the use of scaffolds [4]. Scaffolds can be described as a three-dimensional (3D) structure designed to support 3D defects and guide the complex process of tissue regeneration [5,6,7]. In addition, it is important that desirable characteristics such as biocompatibility, biodegradability, appropriate mechanical properties, and release of bioactive molecules be found in the scaffolds for periodontal tissue repair and regeneration [5,6,8].

The utilization of nanofibrous membranes/scaffolds has become increasingly compelling nowadays [3,9,10]. Electrospinning technology stands out as one of the methods for fabricating these scaffolds, offering attractive features such as low cost and simplicity. This enables the large-scale production of nanofibrous membranes with porous microstructures [10]. This technique involves the use of polymeric solutions (synthetic and natural polymers) that are electrified via an electrohydrodynamic process to generate a jet, which is then stretched to produce the fibers [3,11].

Polycaprolactone (PCL) is a semi-crystalline synthetic polymer known for its considerable biocompatibility and mechanical strength, though it exhibits a relatively slow degradation rate and hydrophobic nature [12]. On the other hand, gelatin methacryloyl (GelMA) is a semi-synthetic polymer extensively utilized in biomedical applications due to its promising properties such as biocompatibility, biodegradability, hydrophilicity, and tunable physicochemical properties [13,14]. In this regard, in the present study, a polymer blend consisting of PCL and GelMA is employed to harness the advantageous characteristics inherent to both polymers within the scaffold. The association of PCL with other natural or synthetic polymers has been widely explored for tissue engineering applications, aiming to create a new material with the desired characteristics of each polymer employed [15].

Of note, PCL and GelMA have presented limited therapeutic and osteogenic properties. Thus, their association with compounds or molecules that present bio-stimulatory potential is necessary and the cerium oxide (CeO) nanoparticles represent one such candidate. Nanoparticles display distinctive characteristics, such as their electronic, optical, magnetic, and mechanical properties, attributed to their size, which can range from 1 to 100 nm, setting them apart from a bulk material [16]. Cerium, the most abundant rare-earth metal, exhibits the redox state found in CeO_2_ and is recognized in the biomedical field due to their redox properties, being extensively applied for different treatments and fabrication of biosensor devices [16]. Hence, this compound has been integrated into the engineered nanofibers developed in the present study, due to its diverse array of biological effects, which encompass osteogenic potential, antioxidant properties, antibacterial action, anti-inflammatory response, anti-apoptotic activity, and angiogenic stimulation [16,17,18].

Therefore, this study aimed to develop and characterize a blend of GelMA/PCL nanofibers scaffolds associated with CeO using electrospinning technique for periodontal tissue regeneration. The scaffolds were submitted (crosslinked—C) or not submitted (non-crosslinked—NC) to a photocrosslinking process, allowing us to conduct a comparative analysis of the chemical, morphological, mechanical, and biological properties, as well as biodegradation/swelling profile of the designed scaffolds.

## 2. Materials and Methods

### 2.1. GelMA Synthesis

GelMA was synthesized following a previously established protocol [9,19]. In brief, 10% (*w/v*) gelatin type A-sourced porcine skin (Sigma-Aldrich, St. Louis, MO, USA) was dissolved in phosphate-buffered saline (PBS; Gibco, Carlsbad, CA, USA) at 50 °C under stirring conditions (300 revolutions per minute—rpm), followed by addition of methacrylic anhydride (276685, Sigma-Aldrich) for gelatin methacrylation at a rate of 0.5 mL/min at 50 °C and 300 rpm; this solution was submitted to continuous stirring for 2 h. After this period, pre-heated PBS (50 °C) was added, and the GelMA was subjected to dialysis against deionized water using a 12–14 kDa dialysis membrane (Spectrum Spectra/Por; Thermo Fisher Scientific, Waltham, MA, USA) for 7 days at 50 °C, aiming to eliminate unreacted methacrylic anhydride and its byproducts. The GelMA solution was then frozen at −80 °C for 48 h, lyophilized, and stored at −80 °C until needed for blend preparation.

### 2.2. Fabrication of Electrospun Nanofiber Scaffolds

**Figure 1:**
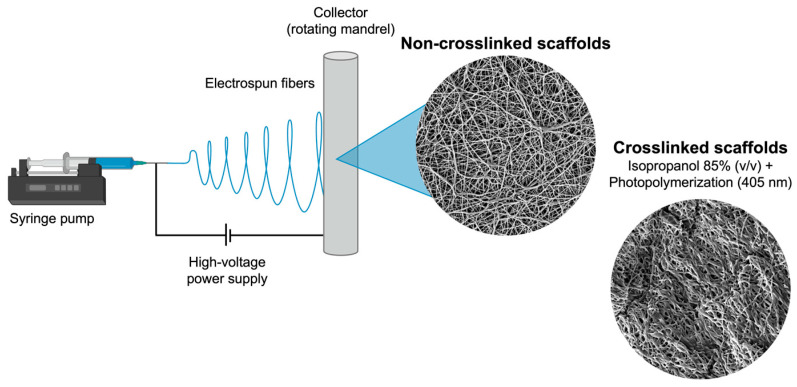
Schematic representation of the electrospinning technique used for the fabrication of the designed scaffolds.

A 15% (*w/v*) GelMA solution was prepared by dissolving it in glacial acetic acid (A491-212, Fisher Chemical, Fairlawn, NJ, USA) overnight for 12 h at 50 °C with continuous stirring at 300 rpm, and 10% of PCL solution (*w/v*) was dissolved in hexafluoro-2-propanol (Sigma-Aldrich) overnight at room temperature (RT) and 300 rpm. Next, an equal amount of GelMA and PCL solution was mixed overnight at 300 rpm at 35 °C to obtain a 50:50 (*v/v*) polymer blend. Then, the crosslinked agent Lithium phenyl-2,4,6-trimethylbenzoylphosphinate (LAP; Advanced BioMatrix, Carlsbad, CA, USA) was added to the GelMA solution at a concentration of 0.075% (*w/v*), which acts as a photoinitiator. For samples containing cerium oxide (CeO; cerium (IV) oxide, nanopowder, 99.5% min (REO), particle sizes of 15–30 nm, Cat. No. 044960.22, Thermo Scientific Chemicals, Waltham, MA, USA), this compound was added at 5% and 10% (*w/w*) concentrations to the GelMA/PCL solutions, stirred for 30 min, submitted to sonication for 40 min and stirred for an additional 20 min in the absence of light. After complete dispersion of CeO to the solutions, nanofibrous scaffolds were fabricated through electrospinning using the following optimized parameters: a 1-inch 25–27 gauge needle, a flow rate of 0.6–0.8 mL/h (KDS101; KD Scientific, Holliston, MA, USA), an applied voltage of 20–22 kV (ES50P-10 W/DAM, Gamma High-Voltage Research, Inc., Ormond Beach, FL, USA), a 15 cm gap between the needle and collector, a rotating collector set to 120 rpm, 18% humidity, and room temperature (Figure 1).

After obtaining the nanofibrous mats, they were placed into plastic bags sealed with aluminum foil for light protection and dried in a vacuum oven for 48 h at RT to eliminate residual solvents. Then, scaffolds were cut to specific sizes based on the required experiments and separated according to the experimental groups: GelMA/PCL (control group), GelMA/PCL + 5% CeO, and GelMA/PCL + 10% CeO; these groups were submitted (crosslinked—C) or not (non-crosslinked—NC) to the photocrosslinking procedure, which involves applying a wetting agent to the scaffolds, the 85% isopropyl alcohol (*v/v*) (2-propanol; A451-5, Fisher Chemical), lightly drying them with low-lint Kimwipes papers (Kimberly-Clark, Irving, TX, USA), and photocuring them with a light-emitting diode curing unit (LED, 405 nm, Light Zone II, BesQual, Meta Dental Corp., Glendale, NY, USA) for 3 min every side [9]. 

### 2.3. Chemical Characterization

Two specimens from each group (10 × 10 mm^2^) were obtained and examined using Fourier transform infrared spectroscopy (FTIR-ATR, Nicolet iS50, Thermo Fisher Scientific, Waltham, MA, USA) to evaluate the chemical properties of the scaffolds. The spectra of non-crosslinked (NC) and crosslinked (C) scaffolds containing CeO (0—control group, 5%, and 10%) were assessed, as well as the spectra of pure PCL, GelMA, and CeO powder. Sixteen scans were collected with a spectrum between 4000–500 cm^−1^ and a resolution of 4 cm^−1^. Baseline correction spectra were normalized for analysis [9]. In addition, NC scaffolds were submitted to chemical characterization before and after immersion in PBS for time points of 0, 3, 7, and 10 days.

### 2.4. Morphological Characterization

To evaluate the crosslinked and non-crosslinked fibers’ morphology and diameter, the scaffolds were analyzed by scanning electron microscopy (SEM) (Tescan MIRA3 FEG-SEM, Tescan USA Inc., Warrendale, PA, USA) [9]. Firstly, two samples of each group (10 × 10 mm^2^) were prepared, fixed in metallic devices, and gold-sputtered for 90 s, then analyzed in high vacuum at 7k× magnification (scale bar 10 µm). SEM images were utilized to measure fiber diameters and frequency distribution using ImageJ Software version 1.54j (Wayne Rasband, National Institute of Mental Health, NIH, Bethesda, MD, USA); specifically, 150 fibers were analyzed for diameter across three random areas of the scaffolds. 

### 2.5. Biodegradation and Swelling Characterization

Both tests were conducted according to previously described methodology [9]. For degradation analysis, 10 × 10 mm^2^ samples were prepared (n = 8/group) for each experimental group, then scaffolds were weighed in dry condition (*W*_0_) and immersed into vials containing 2 mL of PBS (Gibco) solution with 1 U/mL of collagenase type A (collagenase from *Clostridium histolyticum*, Sigma-Aldrich) for up to 60 days. The vials were stored at 37 °C and for predetermined times; the scaffolds were dried at room temperature for 24 h and weighed (*W*_d_) again. The PBS/collagenase solution was changed for a fresh one every 2 days to keep the enzymatic activity. The following formula calculates the degradation ratio (Equation (1)):

(1) $$\;Remaining\;mass\;\left(\%\right)=\frac{W_{d}}{W_{0}}\times 100$$

To assess the swelling capacity of scaffolds, 10 × 10 mm^2^ samples were prepared (n = 5/group), weighed in dry condition (*W*_0_), and immersed in PBS (Gibco) for 1, 3, 6, 24, and 48 h. After the different time points, scaffolds were lightly dried with low-lint Kimwipe papers (Kimberly-Clark) and weighted (*W_t_*). Then, the swelling capacity was calculated using the following formula (Equation (2)):

(2) $$\;Swelling\;capacity\;\left(\%\right)=\frac{W_{t}-W_{0}}{W_{0}}\times 100$$

### 2.6. Mechanical Testing

To evaluate the biomechanical properties of the scaffolds, rectangular samples (25 mm × 3 mm) were prepared and either subjected to LED crosslinking (crosslinked) or left untreated (non-crosslinked), following a previously described procedure (n = 5/group). Using a caliper (Mitutoyo Digimatic Caliper; Mitutoyo Corporation, Tokyo, Japan), the thickness of each sample was measured at three distinct positions to obtain an average thickness. Subsequently, uniaxial tensile testing was performed at a crosshead speed of 1 mm/min using an eXpert 5601 machine (ADMET, Inc., Norwood, MA, USA) to assess stress (MPa) vs. strain (%), Young’s modulus (MPa), tensile strength (MPa), and elongation at break (%). 

### 2.7. Culture of Alveolar Bone-Derived Mesenchymal Stem Cells (aBMSCs) and Human Gingival Fibroblasts (HGF)

The biological assays were carried out with alveolar bone-derived mesenchymal stem cells (aBMSCs) and human gingival fibroblasts (HGF), the main cells presented in the periodontal tissue. Cells from passages 3 to 6 were used for the biological experiments. 

aBMSCs, previously harvested and characterized for mesenchymal stem cell markers CD73+, CD90+, and CD105+ [9,20], were donated by Dr. Darnell Kaigler (University of Michigan, School of Dentistry). These cells were cultured in a cell-culture flask (75-cm^2^; Corning, New York, NY, USA) with complete α-MEM (minimum essential media, with +L-glutamine, +ribonucleosides, +deoxyribonucleosides, Gibco) supplemented with 15% fetal bovine serum (FBS; Gibco) and 1% antibiotic solution (penicillin–streptomycin 10,000 U/mL; Gibco), using a humidified incubator (Thermo Fisher Scientific) at 37 °C and 5% CO_2_. 

HGF was purchased from ScienCell Research Laboratories (cat #2620; Carlsbad, CA, USA) and cultured in a cell-culture flask (75-cm^2^; Corning) with complete fibroblast media (ScienCell) containing 2% FBS (ScienCell), 1% fibroblast growth supplement (ScienCell), and 1% penicillin/streptomycin (ScienCell) at 37 °C and 5% CO_2_, following manufacturer’s instruction. 

### 2.8. Cytocompatibility

The cytocompatibility of the designed scaffolds was analyzed by the AlamarBlue assay (Invitrogen) [9]. For this purpose, scaffolds (n = 8/group) were prepared (12 × 12 mm^2^) and submitted or not to the crosslinking procedure. Next, samples with close weight (7 mg on average) were selected, submitted to disinfection with ultraviolet light (for 1 h on each scaffold side), and placed into 24-well culture plates (Corning). Additional disinfections were executed by applying 1 mL of 70% ethanol on the samples for 10 min, washing them twice with sterile PBS (Gibco) for five minutes each. Scaffolds were stabilized on the bottom of the wells using a sterile stainless-steel ring, promoting standardization of the cell culture area (35 mm^2^). Then, complete culture media (according to each cell type) was added to each sample for 30 min. After this period, the media was removed, and aBMSCs and HGF were individually cultured (3 × 10^4^) on the top of the scaffolds. Samples were incubated for 30 min to allow for the cells’ initial adhesion, followed by the addition of 1 mL of complete culture media to the scaffolds. 

Cells were cultured for 1, 3, and 7 days, and at each time point, cell viability analysis by AlamarBlue assay (Invitrogen) was performed. This test relies on the reduction of the resazurin reagent to resorufin, leading to a noticeable color change that correlates with the mitochondrial metabolism of viable cells. Thus, after each timepoint, the culture media was removed and 10% (*v/v*) of AlamarBlue solution in FBS-free media was applied to the samples for 3 h. Then, fluorescence intensity was determined using SpectraMax iD3 (Molecular Devices LLC, San Jose, CA, USA) at 560 nm (excitation) and 590 nm (emission) wavelengths. Values of fluorescence intensity were transformed into percentages, considering the non-crosslinked (NC) GelMA/PCL group on day 1 as being 100%. 

### 2.9. Osteogenic Potential

To assess the osteogenic potential promoted by the fabricated scaffolds, the mineralized nodule deposition was evaluated by the Alizarin Red assay [19]. For this purpose, samples (n = 8/group) were prepared (12 × 12 mm^2^) and selected as previously described, submitted to disinfection procedures, and aBMSCs were cultured (5 × 10^4^) on the top of the scaffolds with complete α-MEM media for 24 h. After this period, cells were treated with osteogenic α-MEM media (complete media supplemented with 100 nM of dexamethasone, 10 mM of β-glycerol phosphate, and 50 mg/mL of ascorbic acid), which was refreshed every 2 days. 

Following 14 and 21 days of culture, samples were treated by fixation in 70% ethanol for 1 h at 4 °C, followed by rinsing in distilled water. They were then immersed in Alizarin Red solution (40 mM, pH 4.2, Sigma-Aldrich) for 20 min under agitation (300 rpm). After five subsequent washes with distilled water, the scaffolds were air-dried overnight at RT. They were then examined using a stereo microscope (ZEISS Stemi 508; Zeiss, Oberkochen, Germany) and photomicrographs of the scaffolds’ surface were obtained. To dissolve the nodules formed, a 10% hexadecylpyridinium chloride monohydrate solution (*w/v* in PBS) at 37 °C was added to the samples for 1 h under agitation (300 rpm). Scaffolds were disrupted using a forceps, the supernatant was retrieved, and its absorbance was measured at 570 nm (SpectraMax iD3). Cell-free scaffolds submitted to the same experimental procedures as cell-seeded scaffolds were used as blanks to eliminate scaffold background. Values of absorbance were transformed into percentages, considering the non-crosslinked (NC) GelMA/PCL group at day 14 as being 100%. 

### 2.10. Collagen Analysis

Sircol collagen assay was performed (Biocolor, Carrickfergus, UK) to assess the potential of the scaffolds to stimulate collagen production by HGF, the key cells involved in this function [21]. For this purpose, samples (n = 6/group) were prepared (12 × 12 mm^2^) and selected as previously described, submitted to disinfection procedures, and HGF were cultured (5 × 10^4^) on the top of the scaffolds with complete fibroblast media for 24 h. After this period, cells were treated with serum-free fibroblast media, and, after 72 h, cell supernatant was collected and stored at −20 °C. This supernatant was mixed with Sircol dye reagent, incubated for 30 min at 400 rpm, and centrifuged (13,000× *g* for 10 min) to promote the precipitation of collagen molecules linked to the dye. Then, samples were washed with an acid–salt reagent, and centrifuged (13,000× *g* for 10 min), followed by the addition of an alkali reagent to promote collagen-bound dye release. Samples absorbance was assessed at 556 nm (SpectraMax iD3) and serum-free fibroblast media was used as a blank. The concentration of collagen in each sample was calculated with a standard curve. Values of absorbance were transformed into percentages, considering the non-crosslinked (NC) GelMA/PCL group as being 100%.

### 2.11. Statistical Analysis

The statistical evaluation of data was performed using the GraphPad Prism 10.0 computer program (GraphPad Computer program, Inc., San Diego, CA, USA) and Microsoft Excel software version 16.85 (Microsoft, Redmond, WA, USA). Data for mechanical testing, cell viability, mineralized nodules deposition, and collagen synthesis were tested for data distribution, using the Shapiro–Wilk test, and homogeneity, using the F-Test two-sample for variances. Assuming normal data distribution, one-way ANOVA (for collagen production) or two-way ANOVA (for mechanical testing, cell viability, and mineralized nodules deposition) were selected; moreover, the multiple comparisons were determined by means of Sidak and Games–Howell post-hoc underlying assumptions. Fiber diameter data did not present normal distribution (Kolmogorov–Smirnov); thus, non-parametric statistical Kruskal–Wallis, followed by Dunn’s post-hoc test were used for data analysis. Data regarding swelling/degradation ratio were submitted to analysis using confidence intervals. A level of significance of 5% was selected for all data analyses.

## 3. Results

### 3.1. Chemical Characterization

**Figure 2:**
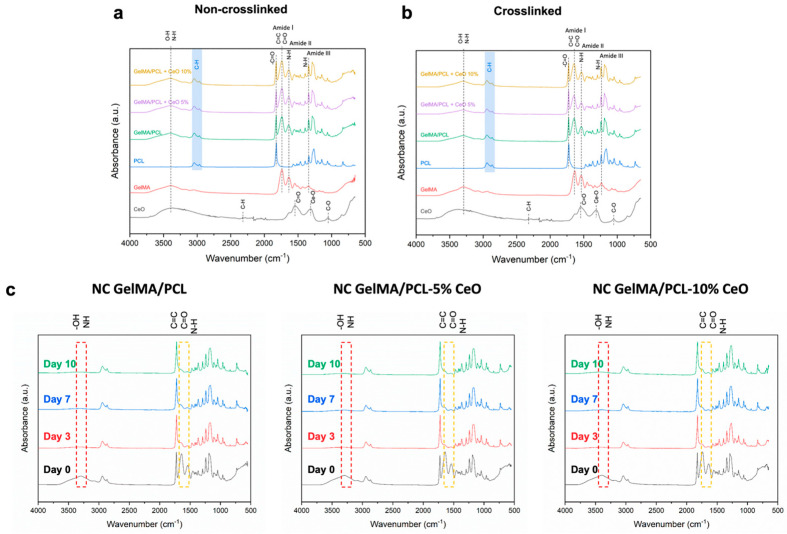
Fourier transform infrared (FTIR) spectra of the non-crosslinked (**a**) and crosslinked (**b**) GelMA/PCL nanofibrous scaffolds containing different concentrations of cerium oxide (CeO) (0–control; 5, and 10% *w/w*). FTIR spectra of the formulated scaffolds immersed in PBS after 3, 7, and 10 days (**c**).

FTIR analysis was performed for the chemical assessment of GelMA/PCL scaffolds before and after photocrosslinking as well as in combination with different concentrations of CeO nanoparticles, as presented in Figure 2.

The chemical composition of the designed scaffolds confirmed the successful blend of the polymers, with merged characteristics peaks of GelMA and PCL, including asymmetric and symmetric C-H (∼2944 and 2866 cm^−1^, respectively), vibration at ∼1723 cm^−1^ showing C=O stretches at PCL, vibration at wavelength of ∼3297 cm^−1^ confirming O-H/N-H stretching, and at ∼1642 cm^−1^ presenting C=O stretching (amide I)/C=C stretching of the methacrylate groups. For GelMA, the N-H bending (amide II) peak was detected at approximately 1535 cm^−1^, and the N-H bending (amide III) peak appeared at around 1235 cm^−1^.

The spectra of pure CeO showed characteristic peaks of C-H stretching (2323 cm^−1^), asymmetric stretching of C=O bond (1546 cm^−1^), Ce-O stretching (1310 cm^−1^), and C-O (1053 cm^−1^); however, in the scaffolds, these characteristic peaks of CeO were overlapped with the peaks of PCL and GelMA (Figure 2a,b).

The chemical composition of the NC scaffolds was also analyzed after their immersion in PBS at different time points, as shown in Figure 2c. It was observed that, after soaking the samples in PBS for 3, 7, and 10 days, the main peaks of GelMA (including O-H/N-H, C=O/C=C, and N-H) were markedly unpronounced compared with the initial time point (day 0) before immersion in PBS.

### 3.2. Morphological Characterization

**Figure 3:**
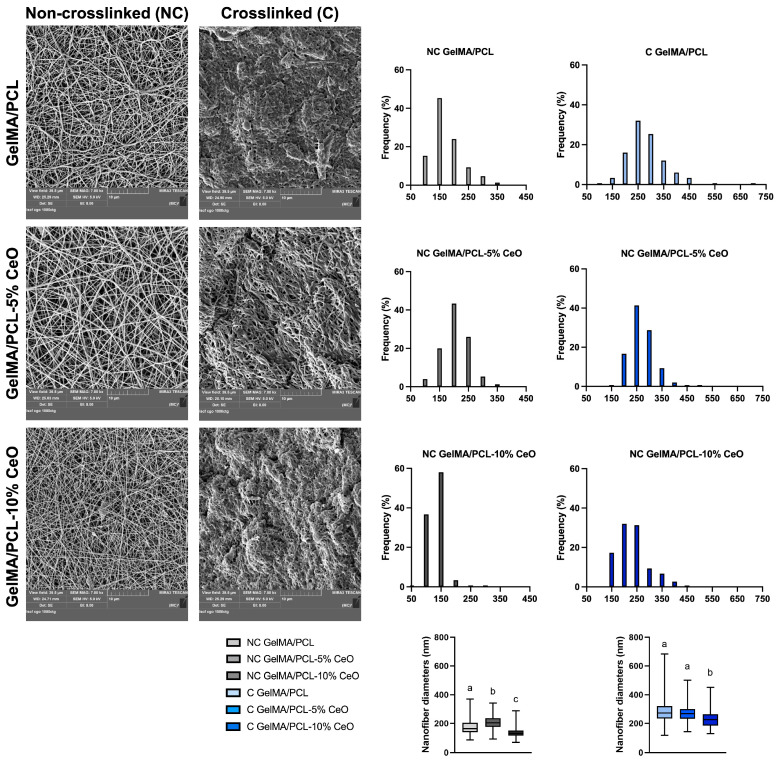
SEM images captured from the surfaces of non-crosslinked and crosslinked scaffolds at a magnification of 7000×. Histograms represent the frequency distribution (%) of fiber diameters studied from 150 fibers. Boxplots show the median fiber diameter values (25th–75th percentiles). Groups identified by different letters show a statistically significant difference (Kruskal–Wallis, followed by Dunn’s post-hoc test, *p* < 0.05).

The fiber topography significantly influences initial cell behavior, including cell adhesion and proliferation. Figure 3 displays scanning electron microscope (SEM) micrographs of both crosslinked and non-crosslinked electrospun GelMA/PCL combined with varying concentrations of CeO nanoparticles. Additionally, the figure illustrates the fiber diameters’ distribution and their median values.

The morphological characterization of the electrospun scaffolds revealed a porous network with randomly oriented nanofibers. NC GelMA/PCL scaffolds demonstrated a high frequency of 150 nm fibers (45%), while NC GelMA/PCL scaffolds incorporated with 5% and 10% of CeO demonstrated a high frequency of 200 nm (43%) and 150 nm (58%) fibers, respectively. For C GelMA/PCL scaffolds, a high frequency of 250 nm fibers (32%) was observed, while for the C GelMA/PCL scaffolds containing 5% and 10% of CeO, the frequencies were 41% of 250 nm fibers and 32% of 200 nm fibers, respectively. For NC scaffolds, the group containing 5% presented the highest diameter of nanofibers (*p* < 0.05) in comparison with the other groups. For C scaffolds, GelMA/PCL and GelMA/PCL-5% CeO showed similar nanofiber diameters (*p* > 0.05); however, GelMA/PCL-10% CeO presented the lowest nanofiber diameters in comparison with the other groups (*p* < 0.05) (Figure 3).

### 3.3. Biodegradation and Swelling Properties

**Figure 4:**
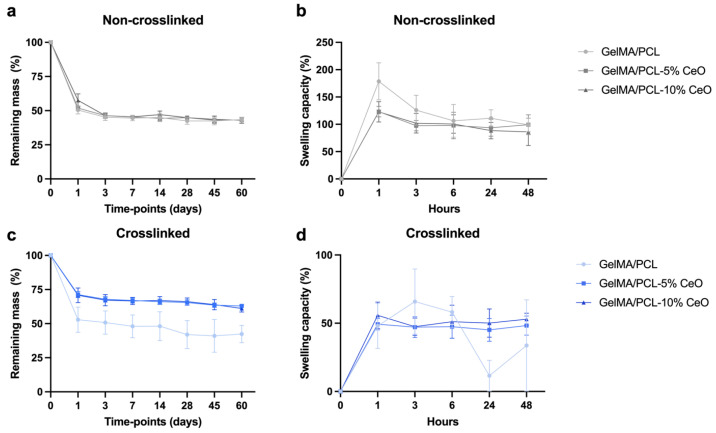
Degradation profile (**a**,**c**) (remaining mass; n = 8) in PBS containing 1 U/mL collagenase and swelling capacity (**b**,**d**) (n = 5) in PBS at 37 °C of non-crosslinked (NC) (**a**,**b**) and crosslinked (C) (**c**,**d**) GelMA/PCL scaffolds containing CeO (0—control; 5, and 10% *w/w*) at different timepoints. Geometric symbols are means, and error bars represent 95% confidence intervals (α = 5%).

All of the formulated NC scaffolds lost ∼50% of their mass after 1 day of degradation analysis, while between days 3 to 60, the loss of mass was slow and continued, resulting in ∼60% mass loss at day 60 for all groups. For C scaffolds, at day 1, the GelMA/PCL group lost ∼50% of their mass, while scaffolds containing 5% and 10% of CeO presented slowed degradation showing ∼30% of mass loss. C scaffolds showed steady and continued degradation between day 3 and 60, with the GelMA/PCL group presenting ∼60% mass loss at day 60 and scaffolds containing CeO, a mass loss of ∼40% at the same timepoint (Figure 4a,c).

Regarding the swelling capacity, in general, NC scaffolds presented a higher swelling capacity than C scaffolds. For NC scaffolds, GelMA/PCL group presented higher swelling capacity over time compared with the other groups, showing the peak of swelling after 1 h (∼180%), while scaffolds containing 5% and 10% of CeO also showed a peak of swelling after 1 h (∼123%). After 48 h, a steady profile was observed, showing swelling ratios of ∼98% for GelMA/PCL, ∼99% GelMA/PCL-5% CeO, and ∼86% for GelMA/PCL-10% CeO. For C scaffolds, a peak of swelling capacity was observed after 3 h for the GelMA/PCL group (∼65%), while for scaffolds containing 5% and 10% of CeO, a peak of swelling was observed after 1 h (∼49 and ∼55%, respectively); after 48 h, a steady profile was observed for CeO-laden scaffolds, showing swelling ratios of ∼48% GelMA/PCL-5% CeO, and ∼52% for GelMA/PCL-10% CeO. For C GelMA/PCL after 24 h, a swelling ratio of ∼11% was observed and, after 48 h, a swelling ratio of ∼33% was observed (Figure 4b,d).

### 3.4. Mechanical Properties

**Figure 5:**
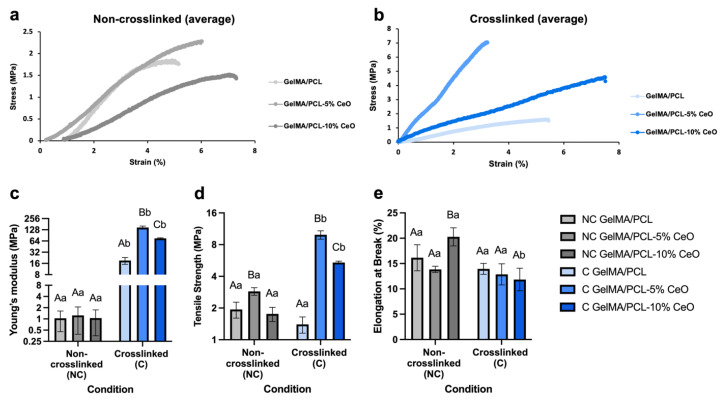
Mechanical characterizations of the designed scaffolds. (**a**,**b**) Stress–strain diagram, (**c**) Young’s modulus (MPa), (**d**) tensile strength (MPa), and (**e**) elongation at break (%) under non-crosslinked (NC) and crosslinked (C) conditions. Columns represent mean values, and error bars represent standard deviations. Statistically significant differences between groups within each condition are denoted by different capital letters, while significant differences between conditions within each group are indicated by different lowercase letters. Analysis was conducted using two-way ANOVA, followed by Sidak’s post-hoc test, with a significance level set at *p* < 0.05.

The biomechanical properties of the scaffolds were assessed using a uniaxial tensile test. Figure 5 shows stress–strain behavior, Young’s modulus, tensile strength, and elongation at the breakpoint of electrospun fibers.

The stress–strain diagram demonstrates that, among NC scaffolds, the incorporation of CeO to 5% affected the mechanical properties positively once the scaffolds showed lower deformation in response to the applied stress. Notably, and regarding the NC scaffolds, we observed initial deformations when installing the samples into the ADMET machine due to their fragility. Consequently, the measurements did not start from the zero point, and it took a few seconds for the machine to accurately assess the mechanical properties, including stress and strain (Figure 5a). Our findings indicate that scaffold stiffness and elasticity do not strictly correlate with CeO dosage. Among C scaffolds, GelMA/PCL-10% CeO and GelMA/PCL showed the highest elasticity under applied stress, followed by GelMA/PCL-5% CeO (Figure 5b). In contrast, for the NC scaffolds, the GelMA/PCL scaffolds displayed a higher Young’s modulus and lower elasticity compared with the CeO-containing scaffolds (Figure 5a), likely due to the impact of cross-linking. All C scaffolds presented a significantly higher (*p* > 0.05%) Young’s modulus compared with the NC scaffolds, with the C GelMA/PCL-5% CeO showing the highest stiffness and lowest elasticity among all groups (*p* > 0.05) (Figure 5c). Regarding tensile strength, comparing NC scaffolds, GelMA/PCL-5% CeO presented the highest values (*p* < 0.05) and, among C scaffolds, groups containing 5% or 10% of CeO showed the highest values (*p* < 0.05). Generally, C scaffolds containing 5% or 10% of CeO presented higher tensile strengths than their NC scaffolds compartments (*p* < 0.05) and NC and C GelMA/PCL showed statistically similar tensile strengths (*p* > 0.05), as presented in Figure 5d. The elongation at break analysis showed that NC scaffolds, particularly GelMA/PCL-10% CeO, presented the highest (*p* < 0.05) value in comparison with the other groups. Among C scaffolds, all groups demonstrated a statistically similar profile (*p* > 0.05). Comparing all conditions, no differences between NC and C GelMA/PCL and GelMA/PCL-5% CeO were observed (*p* > 0.05); however, among C scaffolds, increasing CeO to 10% adversely influences the elongation at break when compared with NC scaffolds (*p* < 0.0001) (Figure 5e).

### 3.5. Cytocompatibility

**Figure 6:**
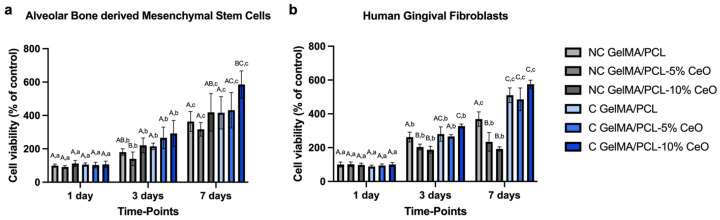
Viability (% of control—NC GelMA/PCL) of alveolar bone-derived mesenchymal stem cells (**a**) and human gingival fibroblasts (**b**) seeded on scaffold surfaces after 1, 3, and 7 days (n = 8) was assessed. Columns represent mean values, and error bars denote standard deviations. Significant differences between groups within each time point are indicated by different capital letters, while significant differences between time points within each group are denoted by different lowercase letters (two-way repeated measures ANOVA, followed by Sidak’s post-hoc test, *p* < 0.05).

The proliferation of aBMSCs and HGF was evaluated by the AlamarBlue assay after 1, 3, and 7 days of cell seeding on different scaffolds. Figure 6 demonstrates the quantitative analysis of the cells’ metabolic activity.

The results show that all scaffolds were cytocompatible over the designated time points. Comparing each group within each timepoint, cells seeded on the scaffolds presented increased metabolic activity after 7 days, except for the group NC GelMA/PCL containing 5% and 10% of CeO. These groups showed statistically similar behavior at days 3 and 7 (*p* > 0.05). In addition, aBMSCs showed the highest proliferation when were seeded on C GelMA/PCL + 10% CeO group (*p* > 0.05) when compared with all groups after 7 days of culture; for HGF, at day 7, C GelMA/PCL and C GelMA/PCL containing 5% and 10% of CeO presented the highest (*p* > 0.05) cell viability when compared with all groups, with no significant differences (Figure 6a,b). 

### 3.6. Collagen Synthesis and Mineralized Nodules Deposition

**Figure 7:**
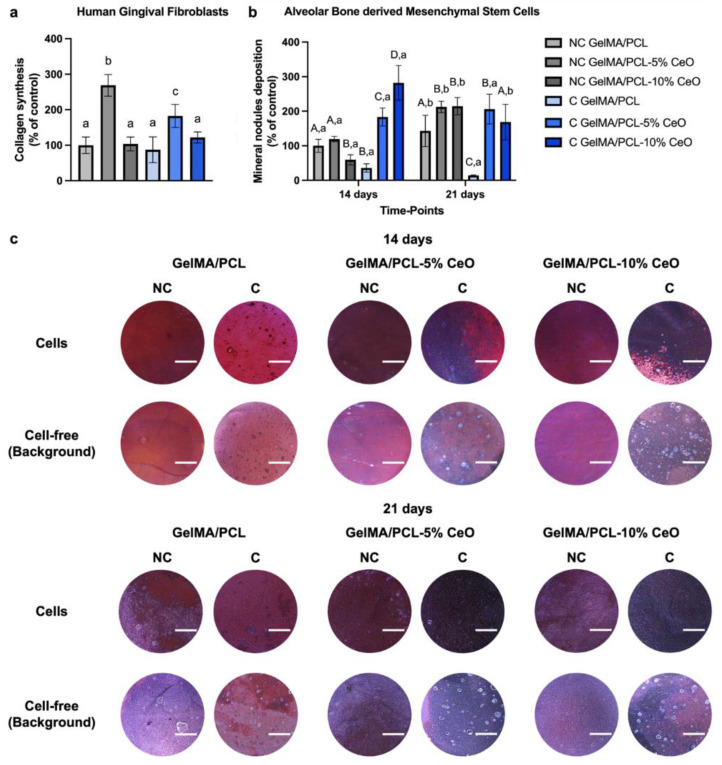
Collagen synthesis (% of control—NC GelMA/PCL) (**a**) by human gingival fibroblasts seeded on the surface of the scaffolds after culturing for 3 days in serum-free media (Sircol assay) (n = 6). Columns depict mean values, with error bars representing standard deviations. Groups identified by different letters are statistically different from each other (Welch’s ANOVA, followed by Games–Howell’s post-hoc test, *p* < 0.05). Quantitative (**b**) (% of control—NC GelMA/PCL) and qualitative (**c**) (scale bar 1000 µm) analysis of mineral nodule deposition (Alizarin Red assay) by alveolar bone-derived mesenchymal stem cells seeded on scaffold surfaces after 14 and 21 days of cell culture (n = 8). Columns represent mean values, and error bars denote standard deviations. Significant differences between groups within each time point are indicated by different capital letters, while significant differences between time points within each group are denoted by different lowercase letters (Two-way ANOVA, followed by Sidak’s post-hoc test, *p* < 0.05).

Collagen synthesis and Alizarin Red assays were used to characterize the HGF functional performance and bone nodule formation of aBMSCs cultured on various electrospun nanofibers. Figure 7a,b show the quantitative analysis of collagen formation and mineralized nodule depositions. Figure 7c presents the images of the Alizarin Red staining of cells seeded on different scaffolds after culturing for 14 and 21 days. The dark red stains (i.e., Alizarin Red staining—calcium chelating product) observed on the scaffolds show the presence of calcium. More positive and darker red staining, typical of calcium deposition, was observed on the CeO-laden scaffold.

The results of collagen synthesis by HGF show that NC or C scaffolds incorporated with 5% of CeO presented significantly higher collagen production in comparison with the other groups (*p* < 0.05). Particularly, NC GelMA/PCL-5% CeO presents the highest levels of this protein production (*p* < 0.05). No significant differences were observed among the remaining groups (Figure 7a).

The osteogenic potential of the scaffolds demonstrates that, at day 14, C nanofibers incorporated with CeO (either 5% or 10%) significantly stimulated mineral nodule deposition by aBMSCs when compared with the other groups of the same timepoint (*p* < 0.05). After 21 days of aBMSCs culture, NC scaffolds containing CeO showed higher levels of mineralized nodule deposition compared with NC GelMA/PCL scaffolds. In addition, C GelMA/PCL scaffolds also presented increased (*p* < 0.05) mineralized nodule deposition when associated with CeO (Figure 7b).

## 4. Discussion

The exploration of nanofibrous scaffolds for GTR represents a burgeoning area of research, underscored by their distinctive attributes that encompass interconnectivity, elevated porosity, and augmented surface area. Leveraging electrospinning technology in the fabrication of these nanofibers not only amplifies their structural resemblance to the extracellular matrix (ECM) but also fortifies their capacity to foster crucial cellular processes such as attachment, proliferation, and differentiation. These processes are pivotal in orchestrating the intricate repair and regeneration mechanisms intrinsic to periodontal tissue [3]. 

This study employed electrospinning technology to fabricate the designed scaffolds, a widely utilized technique for fabricating scaffolds using different polymers that can incorporate bioactive compounds, resulting in nanoscale fibers [22,23]. Electrospinning offers the advantage of creating fibers characterized by a high surface-to-volume ratio, uniform structure, adjustable porosity, and flexibility to conform to various sizes and shapes [23]. However, other fabrication methods, such as 3D bioprinting, can enable the creation of patient-specific spatial geometry, precise microstructures, and the positioning of various cell types to fabricate scaffolds for tissue engineering [24]. More recently, the 4D printing concept has been emerging and involves the incorporation of smart materials capable of controlled and programmed shape changes or property alterations triggered by specific stimuli, such as temperature variations and humidity levels, initiating a transformation process that leads to dynamic and adaptive behaviors [6,25]. Nevertheless, electrospinning has proven to be a robust method for the manufacture of tissue engineering scaffolds due to its cost-effectiveness, versatility, simplicity, and ability to create structures resembling the ECM [23].

Here, the fabrication of GelMA/PCL scaffolds was successfully achieved once the chemical characterization analysis by FTIR demonstrated the main peaks of both polymers within the scaffold, which is in agreement with previous achievements [9]. It has also been reported that CeO nanoparticles show similar wavelengths which explains the overlap of CeO peaks and the polymers [26]. NC scaffolds were subjected to an additional analysis, which involved their immersion in PBS to assess their chemical composition at different time points (0, 3, 7, 10 days), revealing no more sharp peaks for GelMA as initially observed prior to immersion into PBS (day 0). This fact can be explained by the high hydrophilicity and aqueous solubility of non-crosslinked GelMA, leading to low stability in aqueous media and highlighting the importance of crosslinking [13,27,28]. The crosslinking process promotes a polymer nanofiber with enhanced mechanical properties, stabilized 3D structure, and reduced leaching of unreacted components from the scaffolds, which in turn enhances their cytocompatibility, and, taking together, all these factors contribute to the creation of a scaffold that is conducive to cell growth, differentiation, and tissue regeneration [27]. Here, the effects of the photocrosslinking process were assessed.

Similar to the research undertaken by Mahmoud et al. [9], C scaffolds showed higher nanofiber diameters when compared with NC scaffolds. This can be explained by the water absorption from the 85% isopropyl alcohol used as a wetting agent before the photocrosslinking process. The incorporation of CeO into the scaffolds did not affect nanofiber diameters, which can be attributed to the nanoscale dimension of CeO nanoparticles (15–30 nm) and to the adequate mixture/dispersion of this compound before the electrospinning of polymer solutions. All of the scaffolds, regardless of CeO incorporation, showed average nanofiber sizes of 50–400 nm, similar to collagen fiber bundles (50–500 nm), mimicking the native ECM environment [29,30]. The fiber diameters of the designed scaffolds can support cell attachment, proliferation, and differentiation [29,30].

The association of PCL with GelMA could benefit swelling capacity and degradation rate as PCL presents a hydrophobic nature and a slow degradation rate [12], and GelMA presents a hydrophilic nature and faster degradation rate [13,14]. The swelling capacity was higher for NC scaffolds than for C counterparts, which also enhanced the biodegradation. It is known that the crosslinking process results in lower swelling ratios and this fact has previously been demonstrated by Aldana et al., who demonstrated that a higher UV exposure time promoted a higher crosslinking degree and a decreased swelling capacity [13]. 

The incorporation of CeO, in conjunction with photocrosslinking, appears to exert a notable influence on the degradation kinetics of scaffolds containing CeO. This phenomenon can be attributed to several factors: firstly, the presence of CeO within the fibers potentially enhances their stability [31], thereby contributing to a slower degradation rate. Additionally, the photocrosslinking process itself fosters the formation of more resilient fibers, which in turn could be linked to a delayed release of CeO [32] and a consequent deceleration in scaffold degradation. Interestingly, this observed trend was not evident in non-crosslinked (NC) scaffolds containing CeO, likely attributable to the absence of photocrosslinking. This absence may exacerbate the degradation of fibers and subsequently accelerate the release of CeO. Thus, these findings underscore the intricate interplay between CeO incorporation, photocrosslinking, and scaffold degradation kinetics, shedding light on potential avenues for optimizing scaffold design and performance in tissue engineering applications.

The observed augmentation in mechanical properties and concurrent reduction in ductility within C scaffolds can be attributed to the photocrosslinking process, which facilitates the formation of chemical bonds within the polymeric network, thereby bolstering stability [27]. Of note, the introduction of CeO into the nanofiber matrix, as demonstrated by Bianchi et al., did not compromise scaffold mechanical integrity [33]. Rather, the incorporation of CeO resulted in heightened Young’s modulus and tensile strength, indicative of the enhanced stability attributed to CeO’s reinforcement properties. This enhancement is attributed to CeO’s capacity as a nanofiller, effectively occupying void spaces within the polymeric fibers and thereby optimizing stress transfer mechanisms. Consequently, this augmentation leads to superior tensile strength and an elevated Young’s modulus of elasticity, underscoring CeO’s efficacy as a reinforcing agent [33].

The biomechanical properties of periodontal tissues, like alveolar bone and gingiva, vary according to the anatomical site, and differing values are reported in the literature [34,35]. However, it has been demonstrated that the alveolar bone showed values of Young’s modulus of between 70 and 200 MPa [34], while the attached gingival tissue presented values of Young’s modulus of between 19 and 54 MPa [35]. Our designed C scaffolds containing CeO showed values of elastic modulus higher than 70 MPa, which supports scaffold functionality and compatibility with the bone and gingival tissue.

The consideration of cytocompatibility in the scaffold design for guided tissue regeneration (GTR) is paramount, as it directly influences cell proliferation and differentiation [11,36]. In this study, we observed a notably high level of cytocompatibility across all scaffold formulations containing CeO, which supported the viability of both aBMSCs and HGF over time. This enhanced viability suggests potential indirect support for cell proliferation, aligning with previous findings that indicate that CeO stimulates cell viability and proliferation across various cell types, as evidenced by other studies [17,18,37]. The selection of aBMSCs and HGF for biological assays serves to represent the soft and hard tissue components integral to the complex structure of the periodontium [3].

The osteogenic differentiation potential of the designed scaffolds was rigorously evaluated, revealing that CeO-laden scaffolds elicited a significant deposition of mineral nodules by aBMSCs, consistent with findings from various prior investigations [18,38]. It was elucidated that CeO can effectively promote the differentiation of precursor osteoblast cells by activating the Wnt pathway, thereby facilitating the upregulation of osteoblast-related genes [38]. Moreover, scaffolds incorporated with 5% CeO notably augmented collagen synthesis by HGF. Previous studies have indicated that CeO, at specific concentrations, can bolster collagen synthesis both in vitro [39] and in vivo [40]; however, the precise mechanisms underlying this stimulation remain elusive. Notably, fibroblasts, which are pivotal in collagen production within gingival tissue, may be stimulated by CeO, potentially fostering the repair and regeneration of periodontal tissue [41]. Nevertheless, the intricate process of periodontal tissue repair and regeneration necessitates further investigation, particularly utilizing animal models, to comprehensively assess the effects of the developed scaffolds on immune response, angiogenesis, and bone remodeling.

## 5. Conclusions

The CeO-laden GelMA/PCL scaffolds exhibit significant promise as a viable approach for tissue engineering-based therapy aimed at periodontal tissue regeneration. Demonstrating commendable biomechanical properties, biodegradability, and biocompatibility, these scaffolds effectively support the crucial phenotypic attributes of periodontal cells, including proliferation, differentiation, and collagen synthesis, as evidenced by their positive effects on aBMSCs and HGF. This suggests their potential as a robust platform for promoting periodontal tissue repair and regeneration, offering a promising avenue for addressing periodontal diseases and advancing clinical treatment modalities in this field.

## Data Availability

The raw data supporting the conclusions of this article will be made available by the authors on request.

## Abbreviations

aBMSCs, alveolar bone-derived mesenchymal stem cells; α-MEM, minimum essential media; ANOVA, analysis of variance; C, crosslinked; CeO, cerium oxide; CO_2_, carbon dioxide; CP, collagen production; ECM, ectracellular matrix; FBS, fetal bovine serum; FTIR, Fourier transform infrared spectroscopy; GelMA, gelatin methacryloyl; GTR, guided tissue regeneration; HGF, human gingival fibroblasts; LAP, lithium phenyl-2,4,6-trimethylbenzoylphosphinate; LED, light-emitting diode; MND, mineralized nodule deposition; NC, non-crosslinked; PBS, phosphate-buffered saline; PCL, polycaprolactone; rpm, revolutions per minute; RT, room temperature; SEM, scanning electron microscopy; v, volume; w, weight; 3D, three-dimensional.
